# An automated and high-throughput approach for enhanced precision of adenoviral titering

**DOI:** 10.1016/j.omtm.2025.101410

**Published:** 2025-01-20

**Authors:** Paolo Bottega, Manlio Fusciello, Firas Hamdan, Jacopo Chiaro, Salvatore Russo, Federica D’Alessio, Mikaela Grönholm, Vincenzo Cerullo

**Affiliations:** 1 Department of Pharmaceutical Biosciences, University of Helsinki, Faculty of Pharmacy ImmunoViroTherapy Lab, Drug Research Program, Viikinkaari 5E, 00790 Helsinki, Finland; 2 Helsinki Institute of Life Science (HiLIFE), University of Helsinki, Fabianinkatu 33, 00710 Helsinki, Finland; 3 Translational Immunology Program (TRIMM), Faculty of Medicine Helsinki University, University of Helsinki, Haartmaninkatu 8, 00290 Helsinki, Finland; 4 Digital Precision Cancer Medicine Flagship (iCAN), University of Helsinki, 00014 Helsinki, Finland; 5 Department of Molecular Medicine and Medical Biotechnology and CEINGE, Naples University Federico II, 80131 Naples, Italy

**Keywords:** viruses, vaccines titering, vector quantification, oncolytic vaccines, adenovirus, highthroughput microscopy, multiplicity of infection, immunocytochemistry, vaccines, hexon protein

## Abstract

Accurate quantification of viral vectors and vaccines is a crucial step required before any downstream use of virus preparations. The conventional immunocytochemistry-based method of adenovirus quantification has been widely used, but there are many areas for improvement toward accuracy and resource consumption savings to reduce viral miscalculation and wastage of vaccination materials. In this work, a one-step approach is implemented for optimized adenoviral quantification that uses a single antibody coupled with automated, high-throughput image acquisition and subsequent batch analysis. First, cells are infected with the adenovirus of interest and stained using the Hexon protein. Then, multichannel automated image acquisition via the Invitrogen EVOS M7000 Imaging System is performed. Last is an automated large batch analysis of acquired images via the EVOS Analysis Software, accomplished via precise training of the software allowing for virus infection quantification and additional parameters (counts, circularity, area and intensity of targets). It was found that the implemented approach yielded precise and accurate quantification of both oncolytic viral vaccines and gene therapy vectors in a time- and resource-effective manner when compared with conventional methodologies.

## Graphical abstract

**Figure:**
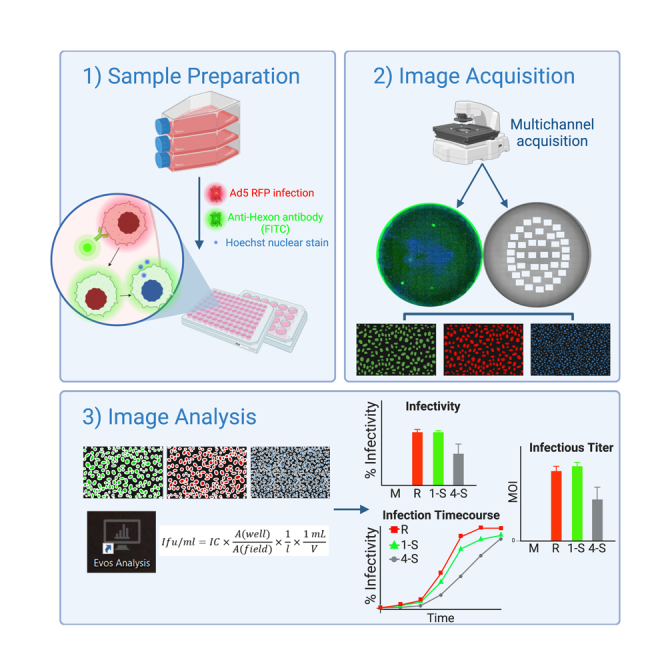

## Teaser

Bottega and colleagues propose an automated, high-throughput adenoviral titering protocol implementing a single antibody system, advanced imaging, and batch analysis. The method improves precision, reduces resource consumption, and enhances the scalability of viral vectors. This innovation provides a transformative tool for vaccine and gene therapy production, offering enhanced efficiency and accuracy.

## Introduction

### Viral vectors and vaccines quantification

The quantification of viral vectors and vaccines is an imperative assessment of the performance and fitness of the biological.^1^^,^^2^ This quantification process can be one that is very taxing on temporal resources, serving as a prominent bottleneck in the viral vector and vaccine production, determining the course and timing for all downstream experimentation.^2^^,^^3^^,^^4^ This encompasses the urgent need to develop and implement accurate and precise methods of viral quantification achievable in a timely manner, accelerating downstream usages notably for dosing *in vitro* and *in vivo*.

This quantification holds central importance in understanding the clinical relevance and clinical dose of viral vectors and vaccines.^5^^,^^6^ In this light, viral titering has been at the forefront of quantifying viral vectors imparting profound insights and understanding the fitness, replicative procedure or cycle of the virus in a cell specific manner.^7^^,^^8^ The fitness of a virus can be defined as the multiplicity of infection (MOI), which is reflective as the ratio of the number of virus particles to the numbers of the host cells.^9^^,^^10^^,^^11^ A focal point in adenovirus quantification are infectious units per milliliter (Ifu/mL) or the amount of infectious viral particles to induce infection in target cells per milliliter.^12^^,^^13^ However, despite the specificity and informative nature of this conventional approach, it is very costly to perform from many variables, most notably in the way of time, finances including reagent and resource costs, and operator requirements.

### The cost-benefit analysis of the current preferred method: Viral titering

**Figure 1:**
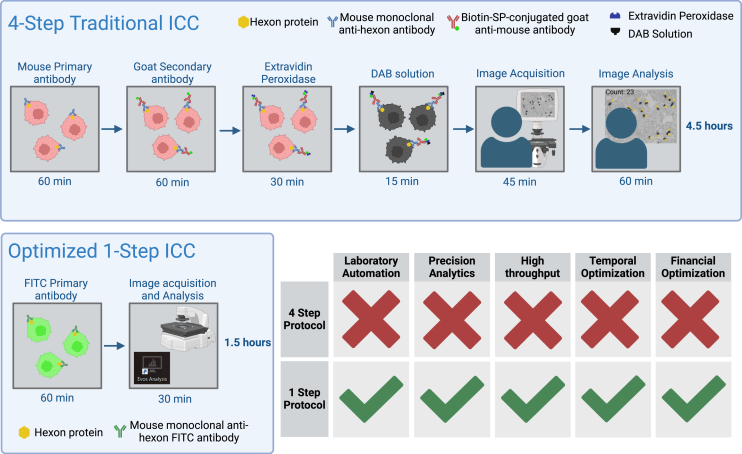
Optimized ICC protocol with associated benefits Benefits include a reduction in resource consumption (temporal, financial, and material), automation and high-throughput analysis, and optimized precision and accuracy. The top demonstrates the traditional ICC protocol comprising of a dual conjugated antibody system requiring sequential administration and manual image acquisition and analysis. This is a low-throughput method with the time required for image acquisition and analysis increasing exponentially with the number of data points. The bottom encompasses the proposed optimized protocol comprising a single antibody system, automated image acquisition, and large-scale batch analysis. This is a high-throughput method with the ability to acquire and analyze large datasets in a timely manner, resulting in more precise, accurate, and reproducible viral quantifications. It should be noted that each arrow is representative of washing steps performed in triplicate, which also greatly contribute to the time necessary to execute the viral quantification process. Image created via BioRender.

The current practice of viral titering involves the use of immunocytochemistry (ICC), which is applied via conjugation of probes to antibodies against the viral Hexon protein.^14^^,^^15^^,^^16^ Hexon is a late gene expressed after viral infection, serving vital roles as a packaging-required protein before host cell burst.^17^^,^^18^^,^^19^ Expression of this protein follows a direct positive correlation with the amount of fully functioning virus in any given viral preparation or downstream experiment.^20^^,^^21^ Conventionally, the detection of Hexon protein requires a dual antibody system comprising of a mouse primary anti-Hexon antibody, which is subsequently recognized by an anti-mouse secondary antibody with Biotin SP-conjugated probe (Figure 1). Substrate addition culminates in the production of a colored precipitate that can be spectrophotometrically quantified, determining the amount of Hexon protein and thus the amount of functional virus in a vector or vaccine preparation.

Despite the specificity of ICC for viral titering, the method itself leaves many areas of improvement both in terms of accuracy and resource consumption in all facets. Viral titering via this conventional method is demanding with respect to the required allotted time for the user, as well as the costs and associated implementation of reagents for its successful completion. This study examines a one-step protocol featuring automated imaging acquisition and analysis, aiming to improve from the conventional titering method without any compromise to the accuracy or precision of this pivotal quantification procedure.

### Rapid and automated high-throughput approach to virus quantification

In this work a high-throughput approach is presented, whose implementation stands to significantly increase the speed, precision, and accuracy of viral titering in viral production across both academia and industrial applications. This includes in-depth descriptions of the single antibody system (fluorescence isothiocyanate [FITC] conjugated), implementation of direct fluorescent reporter gene quantification verification, and state of the art automated image acquisition and analysis via implementation of the EVOS M7000 Imaging system by Thermo Fisher Scientific. This optimized approach stands to limit resources expenditure while enhancing precision and accuracy by mitigating varying forms of bias in the conventional ICC, including user and sampling biases. The accuracy, precision, and applicability of this protocol to different viral vectors and vaccines alike is highlighted in this work via quantification of both oncolytic vaccines and gene therapy vectors.

## Results

### Establishing an optimized method of viral titering

**Figure 2:**
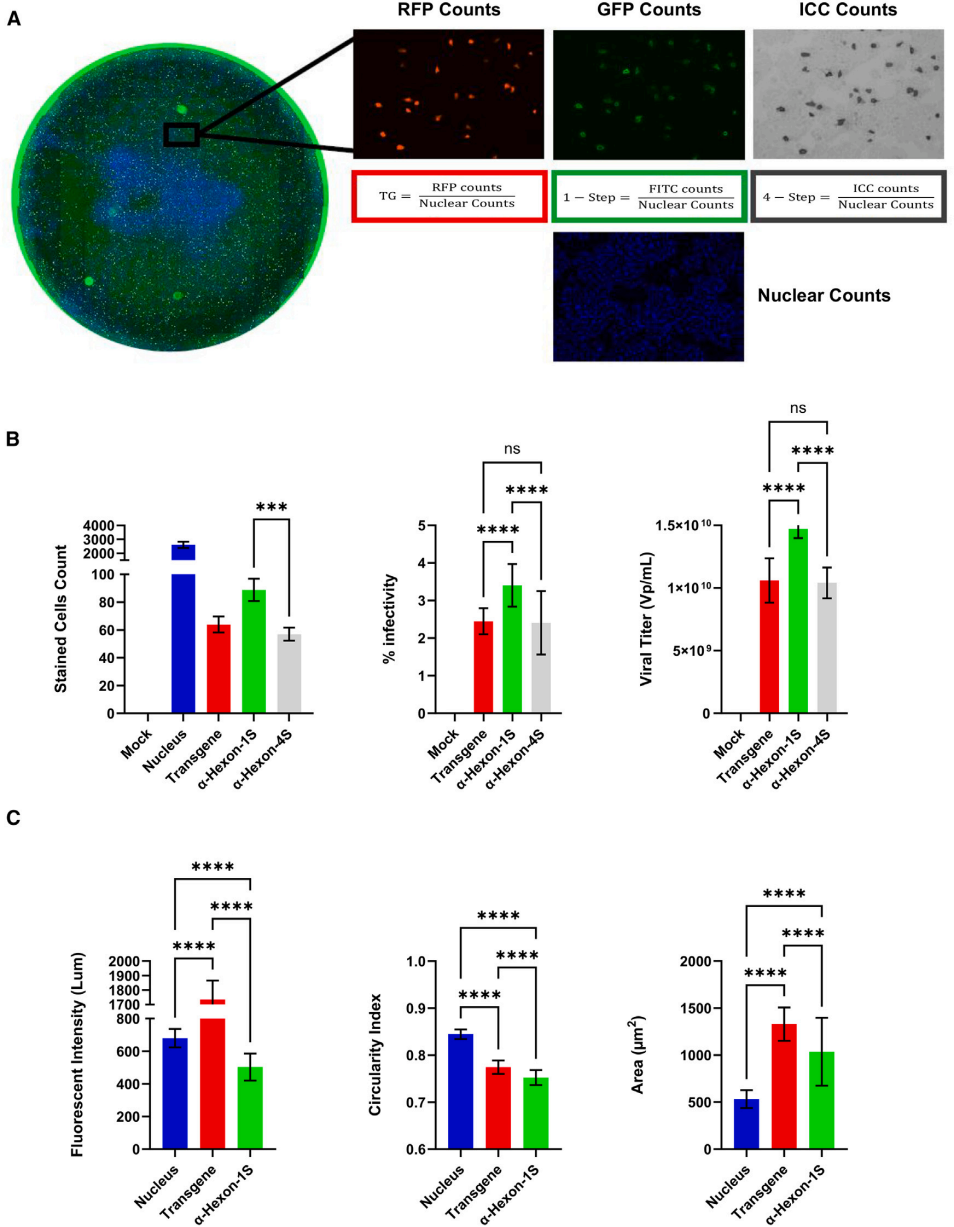
Multi-channel automated image acquisition following specific staining A549 cells were infected with MOI 100 of Ad5D24 for 48 h and imaged. Implementation of nuclear staining method in tandem with FITC single antibody system for a more accurate titering process as well as the determination of percent infectivity with the nuclear stain Hoechst serving as the estimate of total cells and the FITC serving as the estimate of total infection. (A) Acquired data parsing based on microscopy channel, separating the multichannel whole well images to component respective channels. The Hoechst stain (nucleus) acts as an estimate of the total number of cells while the other respective stains represent infection. Mock is uninfected cells across all channels. Transgene (TG) is the direct measure of RFP trans gene production, novel anti-Hexon one-step (1-Step) is the measure of Hexon protein present via FITC fluorescent label, and anti-Hexon four-step (4-Step) is the conventional method by colored precipitate production in the transmitted channel. The counts are obtained for each of the fluorescent stains with the EVOS analysis software while the conventional ICC method requires implementation of the Celleste Software (Version 6) for some form of automated counting or ImageJ manual counting. (B) Individual channel analysis involves manipulation of target counts, via Equation 2 (materials and methods), compared with overall cell counterparts, creating the infection percentage metric. From whole well images highly accurate infectious titer is determined via Equation 2. (C) Other quantifiable metrics from the instrument include the intensity, circularity and area of each spot, all of which tell valuable information about the infectious state of those targets. Images were obtained using EVOS M7000 Imaging System in manual acquisition operation protocols (*n* = 40 images per well, 3 replicate wells per condition). Statistical analysis performed was an ordinary one-way ANOVA with SD reported between wells. Demonstrated is the mean ± SEM and significance levels set at ∗p < 0.05, ∗∗p < 0.01, ∗∗∗p < 0.001, and ∗∗∗∗p < 0.0001 (Confidence interval 95%).

In this study, the synergistic effects of two potential optimizations to the conventional method of titering viruses were investigated: (1) using a one-step anti-Hexon antibody system and (2) implementing advanced microscopic and analytical techniques. As a proof-of-concept evaluation, human lung carcinoma cell line A549 cells, plated at 70% confluency, were infected with in house-cloned Ad5D4RFP and stained; the resulting cells were imaged 48 h after infection. The implementation of this virus serves to detect transgene expression as a direct comparison with Hexon protein staining for the detection and quantification of viral infection. Images were acquired in multiple channels simultaneously and deconvoluted to separate files, each per channel for separate analysis (Figure 2A). Taking each channel separately or combined for analysis allows for a wide array of applications and means of highlighting different trends in the datasets. By using Hoechst staining as a baseline of the total number of cells, the number of cells detected either by FITC or by Red Fluorescent Protein (RFP) was divided by the total number of cells giving an accurate evaluation of the infection process. Direct division of these values in Equation 1 and seen in Figure 2A provides the percentage of infection, a powerful metric that, in tandem with the titer, shows a more accurate measure of the potency of the virus and allows for more accurate experimental design. Building off the total infection counts, percentage infectivity, and titer seen in Figures 2B, a plethora of additional measures are attainable in this form of analysis, which shed further valuable light on the potency of the virus and the progression of infection in general. Metrics such as intensity, circularity, and area are also attained from this analysis at the individual cell level (Figure 2C). The intensity of the stains detecting the virus and circularity of the stained target share a positive correlation with the status of infection, with both metrics increasing as the cytopathic effect (CPE) is observed and as the viral infection progresses. To illustrate this recurring trend throughout this work, a Pearson correlation analysis was performed and summarized in waterfall plots to highlight the correlations existing between the status of infection and fluorescent intensity, circularity and area (Figure S1A). In contrast, the area of the cell shares a negative correlation with the progression of infection as CPE cells are observed to swell up, adopting an increasingly rounded appearance and eventually detach, causing a rapid decrease in the occupied 2D area on the plate.^22^ Enhancements in the current analysis protocol thus allow for a six-metric evaluation of the viral infection, providing a more in-depth view of the progression of viral infection as well as viral potency. Enhanced metrics for assessing viral infection coupled with higher throughput potential results in more accurate viral titering and optimal dosing potential. The anti-Hexon one-step approach yielded higher values in regards to infection metrics, which can be attributed to enhanced accuracy as non-specific binding and background FITC was not observed in uninfected cells.

### Whole-well imaging and titer determination

**Figure 3:**
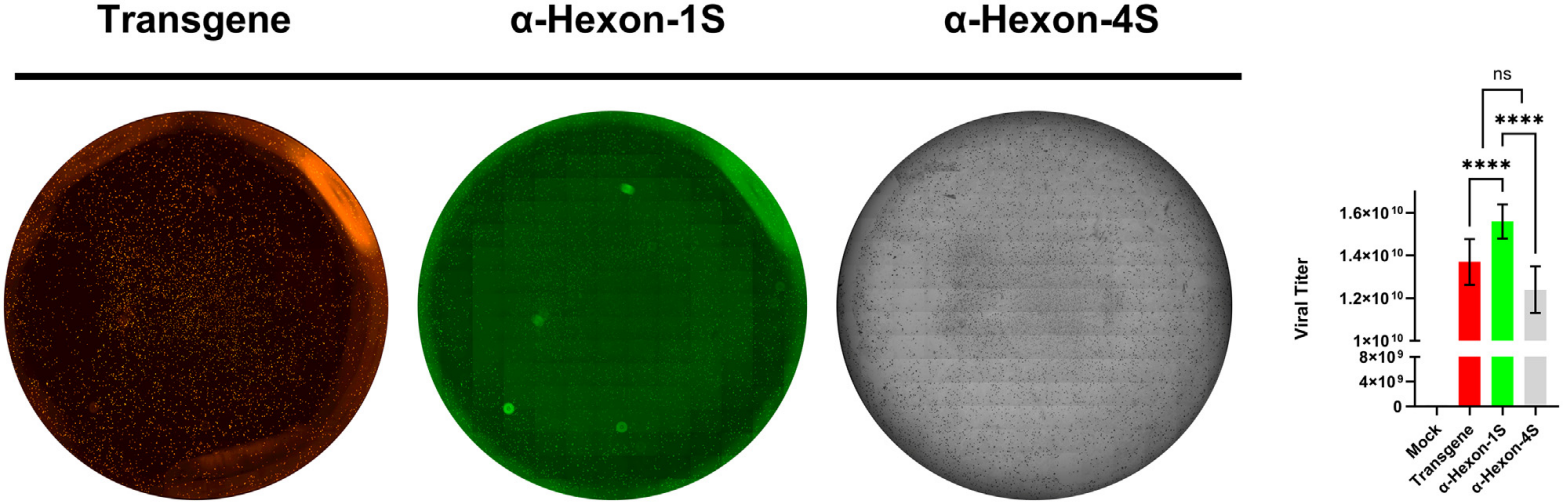
Whole-well imaging for accurate determination of viral titer Multichannel Images (*n* = 192, 3 replicate wells per condition) were simultaneously acquired for a whole standard 24 well, at 10× magnification using the M7000 microscope in automated acquisition operation protocols. Resulting stitched images are generated for each channel separately, and analysis conducted on the individual channel level. Images captured around the edges of the well (containing confluency <50%) were omitted from analysis. Image analysis, including viral titer determination was performed though tandem application of the EVOS Analysis and Celleste Software (Version 6). Statistical analysis performed was an ordinary one-way ANOVA with SEM reported between wells. Demonstrated is the mean ± SEM and significance levels set at ∗p < 0.05, ∗∗p < 0.01, ∗∗∗p < 0.001, and ∗∗∗∗p < 0.0001 (Confidence interval 95%).

Apart from sampling, whole well-imaging served as a powerful protocol for viral titering, enabling the user to consider the full surface of the well in analysis and avoiding distribution and sampling bias. The EVOS M7000 Imaging System is able to acquire high resolution images in a precise and time efficient manner and perform automatized stitching to generate whole well images (Figure 3). Simultaneous acquisition across multiple channels provided data pertaining to the conventional colored precipitate (transmitted), transgene production (RFP) and Hexon production (GFP). This enabled direct titer computation and comparison across the different methods, on the whole well by well basis (Figure 3). The titer obtained by the optimized one-step (optimized) method proved to be highest at 1.56 × 10^10^ and statistically significantly higher than its counterpart methods, suggesting greater sensitivity for signal detection and accuracy of the final titer via this method. A high degree of similarity is reported for the other two methods with reported values of 1.37 × 10^10^ and 1.24 × 10^10^ for transgene and the four-step titering protocols, respectively (Figure 3). In essence, no reported difference could be found when titering the virus according to transgene production of the four-step protocol, while the optimized one-step protocol was found statistically higher.

### Tracking cancer cell killing via oncolytic adenoviruses

**Figure 4:**
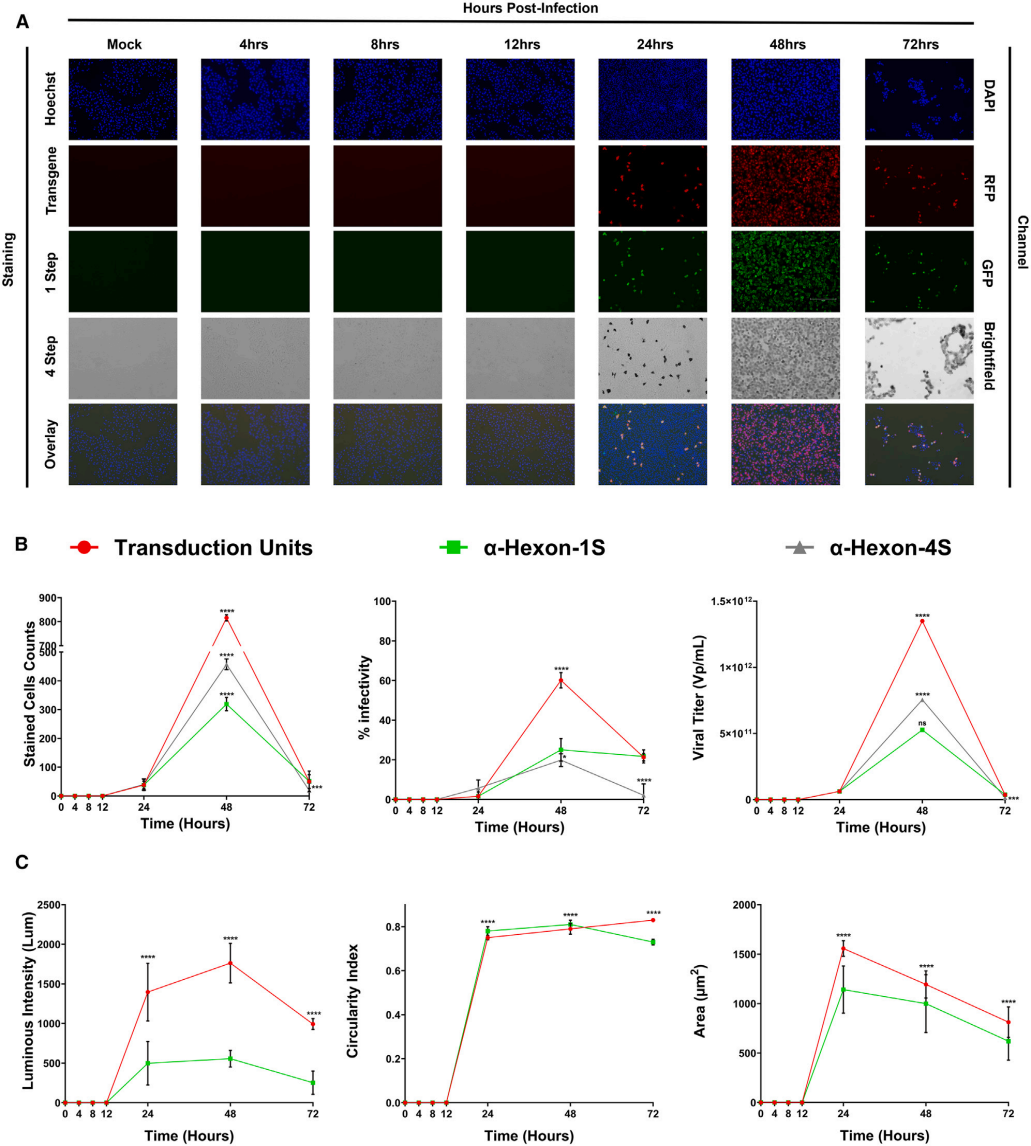
Tracking the progression of oncolytic viral infection across the first 72 h post infection on standard capacity vessel (24 wells) Multichannel images were obtained at landmark intervals according to literature to track the spread of Ad5D24 viral infection (100 MOI) in A549 cells. (A) Full panel obtained in standard 24-well vessel reporting images obtained in the DAPI, GFP, RFP, and transmitted (Trans) channels, and representative overlay of all these channels. Images were obtained using EVOS M7000 Imaging System in automated acquisition operation protocols (*n* = 40 images per well, 3 replicate wells per condition). (B) Metrics obtained from analysis of microscopy imaging demonstrating the viral progression. Counts obtained for each of the fluorescent stains with the EVOS Analysis Software while the conventional four-step ICC method required implementation of the Celleste Software (Version 6) for some form of automated counting or ImageJ for manual counting. (C) Additional measures of viral infection obtained via computations using the previously acquired metrics in (B) and subsequent equations discussed in materials and methods section. Statistical analysis performed was an ordinary two-way ANOVA with SD reported between wells. Demonstrated is the mean ± SEM and significance levels set at ∗p < 0.05, ∗∗p < 0.01, ∗∗∗p < 0.001, and ∗∗∗∗p < 0.0001 (Confidence interval 95%).

Time-based application of the previously discussed methods of acquiring images in multiple channels allows to visualize the spread of viral infection and the subsequent distribution of counts via the different methods over time on a 24-well plate. In adenoviruses, monitoring the first 72 h after infection shed important light into critical time points of infection largely beneficial to all downstream applications and experiments where these or similar adenoviruses are implemented. Implementation of the optimized protocol in tandem with the EVOS M7000 Imaging system rendered this task quite trivial. The first appearances of viral infection are apparent at 24 h with prominent increase of signal from all marker channels registering averaged counts of 38.06, 38.15, and 40.12 for RFP, FITC, and anti-Hexon transmitted, respectively (Figure 4B). The resulting fluorescence intensity was shown to increase steadily over the resulting time points reaching average maximum values of 1762.58 and 497.73L μm for RFP and FITC fluorescent indicators respectively at the 48-h time point (Figures 4A and 4C). This was succeeded by a significant decrease at the 72-h time point due to the detachment of cells from the plate and oncolysis. Subsequent image analysis demonstrated the same trends for infected cell counts, intensity, and circularity of targets with the first appearance occurring at 24 h, maximal effects observed at the 48-h time point before a substantial decrease at 72 h (Figures 4B and 4C). The overarching same trends are preserved in further computed values including the area of targets, the titer and the overall percentage infectivity (Figure 4B). Further, the detection and accuracy of the optimized 1-Step ICC protocol performed comparably to the conventional four-step method showing no statistical significance between critical values such as viral titer which was reported at 48 h maximal values of 5.27 × 10^11^, and 7.55 × 10^11^ for FITC and anti-Hexon transmitted, respectively (Figure 4B). This shows a direct strong positive correlation to the percent infectivity at the 48-h time point being 60.08%, 25.01%, and 19.00% for RFP, FITC, and anti-Hexon transmitted, respectively (Figures 4B and S1B). Taken together, the novel one-step approach was able to perform comparatively with previous methods, shedding more in-depth and accurate data on the first 72 h post adenoviral infection.

**Figure 5:**
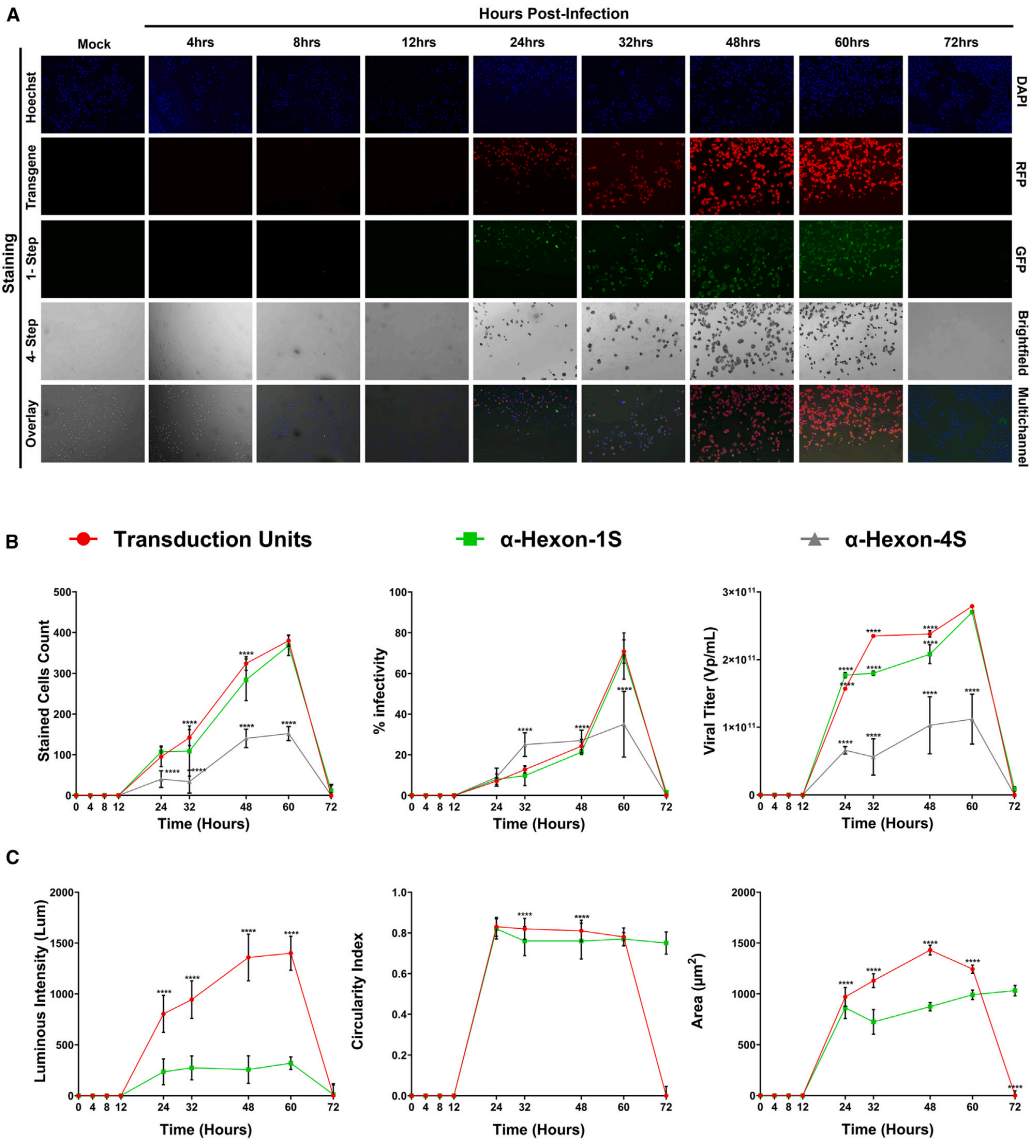
Oncolytic viral infection tracking 72 h post infection on higher capacity vessel (96-well plates) Multichannel images were obtained via the same methods outlined in Figure 4 with notable additional time points implemented in response to the larger capacity of this vessel. (A) Full panel obtained in 96-well vessel reporting images obtained in the DAPI, GFP, RFP, and transmitted channels, accompanied by the respective overlay. Images were obtained using EVOS M7000 Imaging System in automated acquisition operation protocols (*n* = 10 images per well, 5 replicate wells per condition). (B) Metrics obtained using the same parameters as Figure 4 with fluorescent stains analysis in the EVOS Analysis Software and the conventional four-step ICC method via Celleste Software (Version 6). (C) Additional measures of viral infection obtained via computations using the previously acquired metrics in (B) and subsequent equations discussed in materials and methods section. Statistical analysis performed was an ordinary two-way ANOVA with SEM reported between wells. Demonstrated is the mean ± SEM and significance levels set at ∗p < 0.05, ∗∗p < 0.01, ∗∗∗p < 0.001, and ∗∗∗∗p < 0.0001 (Confidence interval 95%).

Repetition of the same experiment was performed on a 96-well plate to exploit the inherent benefits of the vessel (requires lower reagent volumes, allows more wells to allocate more time points, less financially taxing). Similar results were obtained with the onset of viral infection visible at the 24-h time point and marked decrease at the 72-h mark. Increased wells in the vessel allowed to investigate further time points and demonstrated that the maximal signal was achieved at the 60-h time point (Figure 5A). This trend is reflected across the counts and resulting percentage infectivity and viral titers notable with the titers peaking with 60-h values at 2.79 × 10^11^, 2.70 × 10^11^, and 1.12 × 10^11^ for RFP, FITC, and anti-Hexon transmitted, respectively, before marked drops to at or near zero for all methods (Figure 5B). Similar trends were observed again for all methods in regards to intensity, circularity, and area with an increase of the two former parameters over the first 60 h, correlating with a proportional decrease in the latter over that time period (Figures 5C and S1B). Taken together, this eloquently visualizes that it is advantageous to perform this experiment on the 96-well vessel, affording greater time points to elucidate stages of the viral replication cycle with higher accuracy.

To illustrate the versatility of this method specifically for infectivity in normal cells Ad5ddTomato infection of Hek293 cells was used.

**Figure 6:**
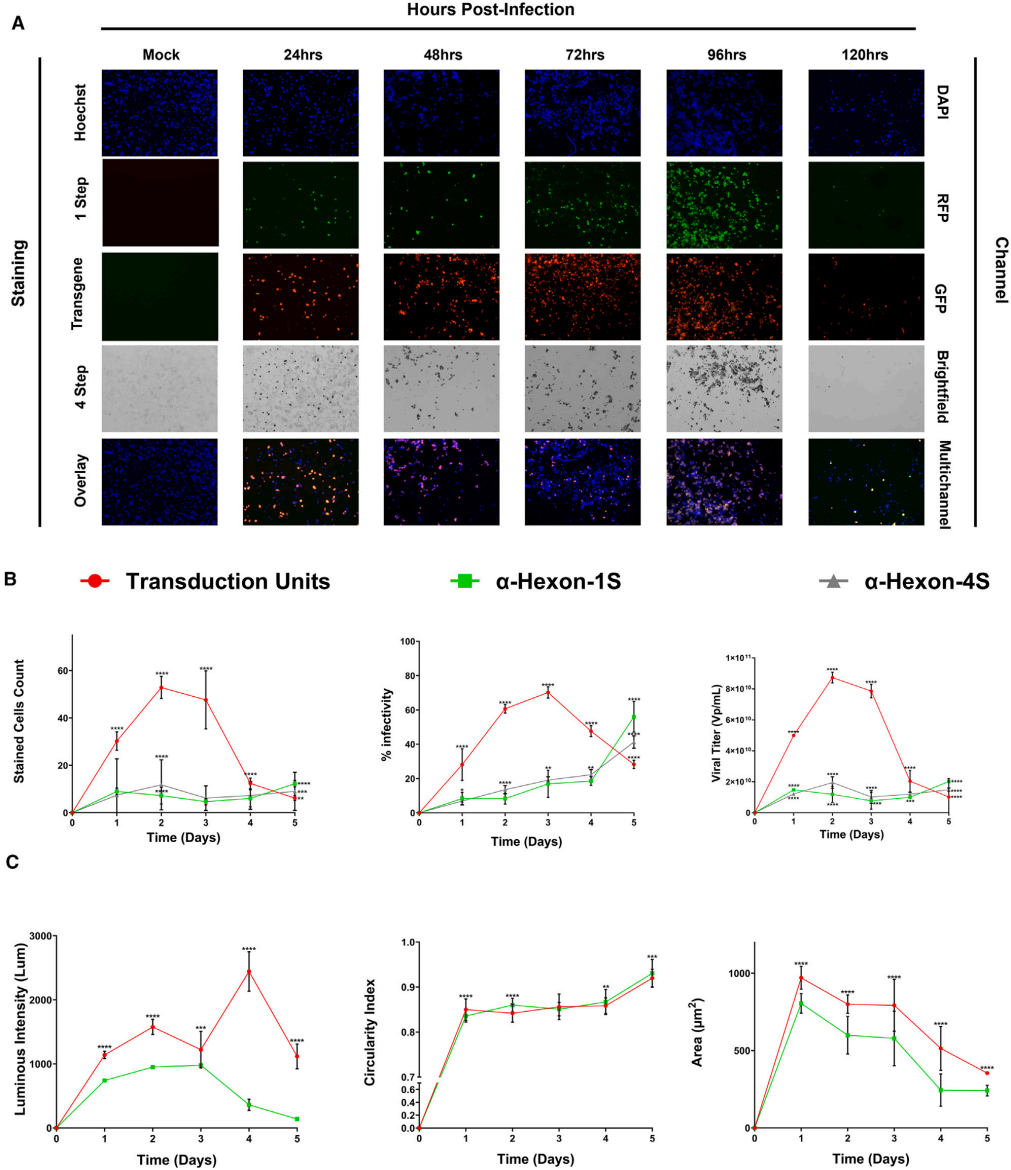
Tracking the progression of non-oncolytic viral infection across the first 5 days post infection on standard capacity vessel (24 wells) Multichannel images were obtained at landmark intervals according to literature to track the spread of non-oncolytic viral infection of Ad5ddTomato. (A) Full panel obtained in standard 24-well vessel reporting images obtained in the DAPI, GFP, RFP, and transmitted channels, and representative overlay of all these channels. Images were obtained using EVOS M7000 Imaging System in automated acquisition operation protocols (*n* = 40 images per well, 3 replicate wells per condition). (B) Metrics obtained from analysis of microscopy imaging demonstrating the viral progression. Counts obtained for each of the fluorescent stains with the EVOS Analysis Software while the conventional four-step ICC method required implementation of the Celleste Software (Version 6) for some form of automated counting or ImageJ for manual counting. (C) Additional measures of viral infection obtained via computations using the previously acquired metrics in (B) and subsequent equations discussed in materials and methods section. Statistical analysis performed was an ordinary two-way ANOVA with SD reported between wells. Demonstrated is the mean ± SEM and significance levels set at ∗p < 0.05, ∗∗p < 0.01, ∗∗∗p < 0.001, and ∗∗∗∗p < 0.0001 (Confidence interval 95%).

**Figure 7:**
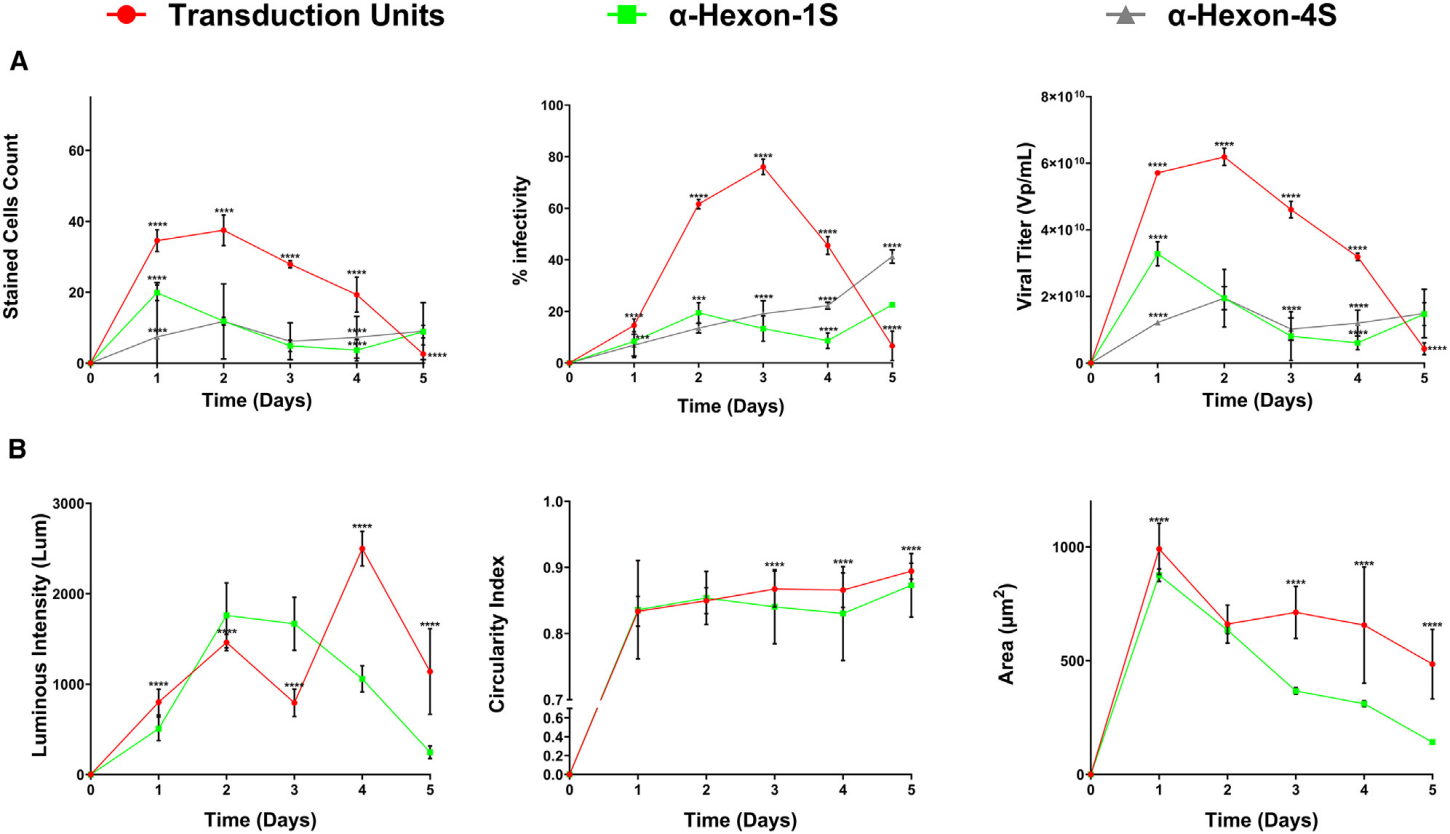
Non-oncolytic viral infection tracking 5 days post infection on higher capacity vessel (96-well plate) Multichannel images were obtained via the same methods outlined in Figure 6. (A) Metrics obtained using the same parameters as Figure 5 with fluorescent stains analysis in the EVOS Analysis Software and the conventional four-step ICC method via Celleste Software (Version 6) or Image. (B) Additional measures of viral infection obtained via computations using the previously acquired metrics in (A) and subsequent equations discussed in the materials and methods. Statistical analysis performed was an ordinary two-way ANOVA with SD reported between wells. Demonstrated is the mean ± SEM and significance levels set at ∗p < 0.05, ∗∗p < 0.01, ∗∗∗p < 0.001, and ∗∗∗∗p < 0.0001 (Confidence interval 95%).

Tracking the replication cycle of Ad5ddTomato yielded similar trends to its oncolytic counterparts; however, given its prolonged replication cycle, measurements were performed once daily over a 120-h period. Onset was observed at the 24-h time point with fluorescent intensities of 1139.44 Lum and 740.38 Lum for RFP and FITC, respectively (Figures 6A and 6C). Throughout the progression of viral infection, the same hallmarks were observed as in the oncolytic platform including, increases in all metrics corresponding to decreasing target area until maximal values occurred marked by titers of 4.99 × 10^10^, 1.48 × 10^10^, and 1.22 × 10^10^ (Figure 6B). Viral maturation and bursting of host cells were again observed on day 5, culminating in significant decreases across many of the computed metrics. Repetition performed on a 96-well plate generated comparable data and trends in accurately quantifying non-oncolytic viruses throughout the replication cycle in its entirety (Figure S1B). This is highlighted by transgene production at the 24-h mark, maximal signal on day 4, and significant decrease in signal corresponding to the completion of the viral replication cycle on day 5 (Figure 7).

### Viral titering vessel comparison

**Figure 8:**
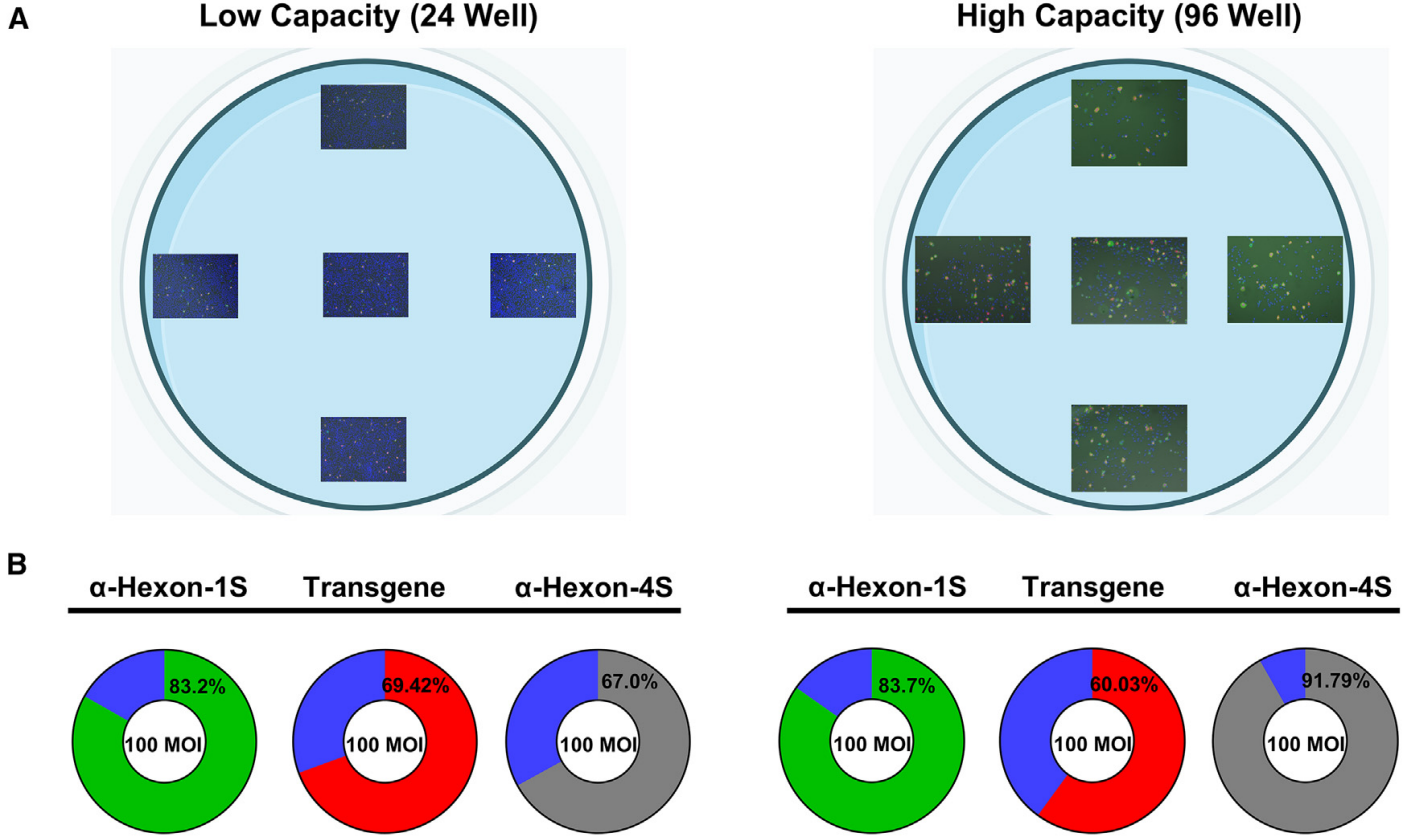
Comparison in the accuracy of percentage infectivity computation across different capacity vessels Measures of the accuracy obtained when using low- and high-capacity vessels for assessing the oncolytic viral infectivity of Ad5D24infection in A549 cells at 100 MOI for 48 h. (A) Schematic of the relative surface area of the well which is captured in five images for low- and high-capacity vessels. Percentage of well surface area computed through vessel manufacturer specifications (24 well, 1.93 cm^2^; 96 well, 0.33 cm^2^) and standard pixel conversion through EVOS analysis scale bar. Image adapted in BioRender ©2024. (B) Resulting percentage infectivity obtained from five multichannel images per well. Image acquisition was performed using EVOS M7000 Imaging System in automated acquisition operation protocols. Fluorescent stain analysis performed on EVOS Analysis Software and the conventional four-step ICC method via Celleste Software (Version 6).

Through tracking of diverse types of viruses over the first 72 h after infection, it became apparent that it is advantageous to implement higher capacity vessels to simultaneously evaluate greater numbers of variables. To fully understand the ramifications this would have on the accuracy of the obtained data with respect to the percentage of infected cells, a complete vessel comparison was performed between the 24-well plate and its 96-well counterpart. Percentage infectivity was the most suitable metric for this comparison, as it gives the most precise estimate of the total number of positive targets which are accurately counted across all different evaluated channels as well as how this accuracy is factored into subsequent calculations such as the percentage infectivity. For each vessel, five images were obtained per well, which encompasses a mere 11.76% of the surface of a well in a 24-well plate, as compared with a staggering 69.0% of the surface of its 96-well comparator (Figure 8A). The ability to achieve such high coverage of the 96-well vessel allows the possibility to acquire images from many more wells and conditions in the same analysis time as very few could be achieved in the 24-well vessel. To ensure this is not at the expense of precision, accuracy was tested as a measure of percentage infectivity of Ad5D24 across three different methods at the optimal MOI of 100. The results are accurately comparable for all methods across the vessels especially at the 100 MOI condition, which is the typical MOI used for quantifying Ad5D24. This is apparent from the detection of some larger number of positive signals and higher percentage infectivity particularly for the anti-Hexon four-step method at 91.79% compared with that of 67.0% in its low capacity counterpart (Figure 8B).

## Discussion

A comprehensive and precise understanding of the specific replication cycle for each virus is crucial for proper production and downstream usage. It has been effectively shown that this optimized method excels at pinpointing specific cycles of viral replication in a resource effective manner. The replication cycle of oncolytic Ad5 viruses is well characterized as a four-stage process with the following stages and approximate occurrence post infection; attachment, entry and endosomal escape (1–2 h), transcription and replication of early gene markers (6–12 h), late gene expression and assembly (12–24 h), maturation, and release (24–72 h post replication) with a total replication time of approximately 72 h.^23^^,^^24^ Conversely, gene therapy vectors such as Ad5ddTomato typically exhibit longer replication cycles culminating over many days until eventual maturation and release of virions. The high temporal variability of replication cycles and activity between different types of viruses demonstrates the critical importance for optimized methods of viral quantification like that of this work. Time course investigation into both Ad5D24 and Ad5ddTomato allowed to capture the optimal times for viral collection right at the conclusion of late gene expression and assembly and before the onset of maturation and release to be at 60 and 96 h, respectively.

The knowledge gained from such an optimized method allows to determine the precise hours for optimal viral harvesting (highest yield and titer), as well as establishing the most accurate titers for correct dosing in downstream experimentation. The ability to scale up to a 96-well plate via this method allows to evaluate a larger number of time points in optimized time given the high-throughput nature of this method given its automated protocols. To this end, the implemented method creates the potential to track viral infection to the precise optimal hour for each and every viral production, a concept unimaginable via the conventional titering methods for the significant resource consumption required to complete such a task, especially with regard to acquisition and analysis times. As such, this method is suitable and a prominent advancement for tracking precise viral infection whether that be in the way of cancer cell killing via oncolytic adenovirus Ad5D24 or gene therapy vector Ad5ddTomato tracking in healthy cells, the applicability and viral quantification options are bountiful with the proposed methodology.

One of the prominent advancements of this method which led to such resource savings was the ability to scale up to a 96-well plate without compromising on the obtained titer accuracy. In assessing the possibility to scale up to a vessel with more testing conditions, the precision and accuracy of the methodology comes into question and was evaluated via the vessel comparison experimentation series. When seeded and infected to appropriate confluency and MOI (100 MOI) the accuracy between vessels was found to be statistically insignificant for all tested gene products. This demonstrates a successful methodology design translatable to higher capacity vessels without compromising the precision and accuracy of titer determination. Conventional ICC methods proved to be unreliable for detection, even at the desirable MOI suffering of target overevaluation in higher capacity vessels. In higher capacity vessels the smaller surface area of each well results in generally darker conditions and less negative space, and using the conventional methods it is difficult to train the software to detect differences between positive targets and negative background, resulting in overevaluation and overall higher percentage infectivities. The inverse phenomenon was observed in the lower capacity vessels, given the greater surface area resulting in positive target undervaluation and lower percentage infectivities. Minimal discrepancies between black and white positive targets and background, respectively, masked by many gray and dark areas in the well is a prominent flaw of the conventional titering method. This culminates in significant challenges when training software to accurately quantify viral infection, and large variability between wells, regardless of the vessel implemented for study. Another prominent effect is the induction of personalized interpretations between different researchers. As observed in this study, the implementation of a fluorescence-based model of detection not only accelerates the process but improves accuracy of the method at 100 MOI, regardless of the vessel in question. Among the investigation of the viral replication and fitness, the optimal range that we have been using is between 10 and 100 MOI. MOI values exceeding this range result in toxicity, while those lower than this range exhibit detection levels below the detection limit of the assay and are not reliable for quantification.

With the ability to scale up to a larger capacity vessel without compromising on accuracy, significant benefits are presented in the ability to test many more conditions on the same plate, while consuming fewer valuable resources from reagents to finances and operator time. This directly translates to the ability to intimately track viral infection across numerous time points in a resource effective manner all within one vessel.

Taken together, the proposed method combining fluorescent titering detection and automated acquisition and analysis offers a plethora of benefits over conventional titering approaches. The most prominent advancements include the ability to provide more comprehensive and accurate viral titering while economizing resource consumption to achieve viral quantification. The former can be credited to many of the advancements in the methodology design, given the high-throughput abilities, allowing for more data collection in a much faster time and resulting in a better and more complete picture of the conditions across the whole surface of the well. This avoids the prominent effects of sampling bias that plague the conventional viral quantification methods and leads to more precise computations and analysis downstream. The ability to consider the full conditions of the vessel, without requiring additional resources, demonstrates the high-throughput nature of this protocol and the profound benefits it offers when quantifying diverse viral platforms.

However, being a titration method based on infectivity it is important to consider that the proposed technology depends both on the cell line used and the experimental conditions implemented, requiring laboratory- and protocol-specific optimizations. As a result, the proposed technology is adaptable to diverse infection methods as it can be applied to diverse cell lines as demonstrated through both A549 and Hek293 implementation in the following work. Nevertheless, cell lines have been chosen in this work according to the desired to be quantified. For instance, have implemented A549 for oncolytic platforms, as they can retain their oncolytic features and it is not advisable to be done in cell lines like Hek293, which via recombination would provide E1 pushing for the occurrence of the wildtype phenotype and loss of oncolytic potential. Furthermore, the proposed method is effective in attaining the Ifu/mL of adenoviruses in a precise and high-throughput manner. This method does not provide other critical information such as the total viral particles, which could be attained by other approaches such as qPCR or RNA probe quantification in tandem with the proposed method.

One limitation of this study lies in the analytics of the additional metrics provided by the EVOS Analysis software, pertaining to circularity and area of positive targets. This analysis is performed on a target by target basis thus even singular targets are sufficient to induce a non-zero measurement (Figures 5C and 6C). This comments to the precision of this methodology and the potential to accurately train the software for detection down to the single cell level, however this can also lead to misleading results. At 72 h for Ad5D24, the amount of viable intact cells remaining on the well is negligeable, as viral maturation and host cell burst has already occurred, an observation not reflected in the area and circularity for this time point, as even singular remaining cells are sufficient for nonzero detection. Even after the full viral replication cycle is completed and host cell burst has occurred in most cells on the plate, the very few cells that remain are detectable for certain metrics of the EVOS system, which can lead to false, non-zero values at these time points. For this reason, at the 72-h time point, other prominent metrics like the titer and the percentage infectivity drop to negligible values, corresponding with cell bursting, while metrics like the area of the cell remain detectable, as even a very small number of cells that have not burst yet is sufficient to generate the same strong signal for these metrics, an important drawback to consider when implementing this protocol.

Further studies are required to optimize software training and the automation analysis protocols to avoid misleading analyses and explore the full potential of this protocol and other applications. For instance, a plethora of cell morphological data is obtained from this methodology, which was used in this work to correlate CPE throughout viral infection as A549 or HEK293 cell morphology demonstrated decreasing area and increasing circularity over time. Tacking the hallmarks of CPE and quantifiably being able to determine viral infection status at all stages of the process is only one potential application for the metric obtained by this methodology. Further studies investigating viral adenoviral infection and the associated prominent morphological effects on neurons, dendritic cells, and macrophages would play a pivotal role in determining the full potential of this methodology and how to harness the most out of the additional metric especially circularity and area of targets.

## Materials and Methods

### Cell lines

The A549 human lung carcinoma cell line and the Hek293 human embryonic kidney cell line were acquired from the American Type Culture Collection (ATCC). These cell lines were maintained in low glucose DMEM (Gibco), supplemented with 10% fetal bovine serum, 1% Glutamax, and 1% penicillin/streptomycin. Cells were seeded to a confluency of between 70% and 80% before all experiments with the higher end of the range employed for the lower capacity 24-well vessels and the reciprocal for higher capacity 96-well vessels. Cell line implementation was selected based on the nature of the vector desired for quantification. We have described A549 for what comes for oncolytic adenovirus, as they are the optimal replication tool for preserving oncolysis through generations; using Hek293 would eventually push the recombination into a wildtype phenotype due to E1 expression of the cell line. Hek293 are advised for normal adenoviral vectors as stated throughout the article. This method has the potential for implementation to quantify diverse adenoviral vectors of varying nature, and careful selection of the appropriate cell line based on said nature is imperative.

### Adenoviruses

Oncolytic adenovirus5D24 (Ad5D24) was produced, propagated, and characterized following conventional adenovirus preparation protocols.^25^^,^^26^ Ad5D24 is an oncolytic adenovirus featuring a 24-bp deletion in the E1A gene and was used for infection of A549 cell line. The virus also boasts an RFP gene whose incorporation into the E3 region was accomplished via integration of monomeric RFP complementary DNA into shuttle plasmid (pTHSN). Subsequently, pAd5D24RFP was produced by facilitating homologous recombination in *E. coli* between linearized pTHSNRFP and linearized pAd5D24. Ad5ddTomato was produced using previously described standard protocols.^27^ Ad5ddTomato is of the serotype 5 with double deletions in the E1 and E3 regions. Transgenes were cloned via replacement of the of the E1 region using Gibson Assembly, as previously described.^28^ In this work five different batches of A54 cells were infected with three different batches of Ad5D24, as well as 3 different batches of Hek293 infected with two different preparations of Ad5ddTomato, demonstrating the batch replicability of the methods.

### Conventional viral titer determination

By conventional method, the viral titer was determined by an ICC assay. This is a four-step protocol establishing the infectious titer based on newly produced Hexon protein after viral infection. Cell fixation was performed with 4% paraformaldehyde (15 min incubation) before staining. A two-step antibody system was employed comprising of primary Mouse Monoclonal anti-Hexon (NB600-413, Novus, 1:2,000, 1 h at room temperature [RT]), and secondary Biotin-SP-conjugated goat anti-mouse (115-065-062, Jackson Immunoresearch, 1:500, 1 h at RT) antibodies. Additional incubation steps were then required for both the enzyme (Extravadin Peroxidase, 1:200, 30 min at RT) and substrate (DAB solution, 1:10, 5 min at RT) solutions. The target cell line is then plated to a confluency of 1.0 × 10^5^ cells/well (24-well plate) and 1.0 × 10^4^ cells/well (96-well plate) and infected with appropriate virus. At 48 h post infection, the above four-step protocol was performed in accordance with manufacturer instructions. The resulting produced colored precipitate allows for microscopic visualization of viral infection in the transmitted channel. After antibody staining, Hoechst nuclear staining (1:1 × 10^5^, 15 min at RT) was performed following standard protocols to establish an estimated total cell count for each condition. Throughout the course of this work, this conventional method served as the benchmark and positive control, establishing the current standard for titering viruses. For the varying time points infection was performed simultaneously for all time points, and cell fixation and staining were performed separately at each desired time point after infection.

### Optimized viral titer determination

MOI and viral quantification were also the relied upon method of determining the titer of a virus in the optimized protocol and the main investigation of this research. The optimized protocol was composed of a single step and single antibody system to again target the newly synthesized Hexon protein after viral infection. Cell fixation was performed in the same manner as the conventional viral titer protocol above. Adenovirus Hexon Monoclonal Antibody, FITC (MA1-7329, Thermo Fisher Scientific, 1:100, 1 h at RT) was used and administered following manufacturer’s protocols. All other protocols including cell seeding, hour after infection of the assay and Hoechst staining were kept consistent with the conventional protocols. The resulting production of FITC was visualized microscopically directly, negating the need for enzyme, substrate systems. For the varying time points, infection, fixation, and staining were executed in the same manner as the conventional viral titer protocol above.

### Microscopy and quantification

Microscopy visualization of viral infection was conducted on the EVOS M7000 Imaging System (Thermo Fisher Scientific) where images of the infected cells displaying the colored precipitate, or FITC were obtained in the transmitted and Green Fluorescent Protein (GFP) channels respectively. Settings including brightness, focus and counts per image were established manually, with the desired targets per image (infected cells) set to between 10 and 100, optimized by selected infection time and MOI in prior stages. After manual calibration automated image acquisition was performed to obtain n images per well across multiple channels. Automated image acquisition for this study included transmitted (for conventional colored precipitate product), RFP (transgene production), GFP (FITC), and DAPI (Hoechst) with 40 images per well for sampling and 192 for whole-well images. Images were obtained in random order and selected randomly in the same locations across all wells for all experiments. The resulting obtained images were also stitched together after acquisition to generate whole-well images of different conditions. The percentage infectivity metric is obtained by dividing the total counts of fluorescent positive cells (from microscopy, RFP as transduction units or GFP as Anti-Hexon) by the total number of cells (represented by nuclear staining (Hoechst staining) as is represented by Equation 1 below and in Figure 2A:

(Equation 1) $$\%\;infectivity=\frac{IC}{TC}\times 100\text{,}$$

where *IC* is the number of infected cells per field and *TC* is the number of total cells.

Conventional data require analysis either manually via ImageJ or via Celleste (Version 6) application (Thermo Fisher Scientific) macro analysis. A novel approach employing the Batch analysis of the EVOS Analysis application (Thermo Fisher Scientific) was used to determine a plethora of parameters related to viral infection on the cells. The process involves training the program to recognize targets from the background and then to apply these learned settings to all images acquired in a dataset to automatically generate the data. Metrics including cell counts, cell area, fluorescence intensity and circularity were automatically obtained for each target by this protocol and exported to a CSV file for further analysis. From this analysis valuable information such as the viral titer was computed using Equation 2:

(Equation 2) $$\frac{Ifu}{mL}=IC\times \frac{A\left(well\right)}{A\left(field\right)}\times \frac{1}{l}\times \frac{1\;mL}{V}\text{,}$$

where *IC* is the number of infected cells per field, the *A(well)* for a 24-well plate is 190 mm^2^, the *A(field)* is the surface area of the field, l is the dilution factor, and *V* is the volume of virus dilution applied per well.

## Data and statistical analysis

Statistical analysis was performed using GraphPad Prism 10.0 Software (GraphPad Software Inc., California USA). All results are expressed as the mean ± SEM and significance levels were set at ∗*p* < 0.05, ∗∗*p* < 0.01, ∗∗∗*p* < 0.001, and ∗∗∗∗*p* < 0.0001 (95% confidence interval). A detailed description of other statistical tests used can be found in the figure title for each corresponding experiment.

## Data and code availability

The authors confirm that all data supporting the findings of this study are available within the article and its supplementary materials.

## Acknowledgments

We would like to acknowledge the contributions of 10.13039/100010116Medicinska Understödsföreningen Liv och Hälsa and 10.13039/501100009067K. Albin Johansson Foundation for financial support throughout this work. We would also like to acknowledge Thermo Fisher Scientific for their technical and material support during the project, especially to Adyary Fallarero, PhD, Stephen Full, PhD, and Mikhail Okun, PhD.

During the preparation of this work, the author(s) used ChatGPT, developed by OpenAI, to enhance the readability and improve the language of this manuscript. After using this tool/service, the author(s) reviewed and edited the content as needed and take(s) full responsibility for the content of the publication.

## Author contributions

P.B., M.F., M.G., and V.C., conceptualized and designed the research. P.B. performed the research investigations and related data analysis with contributions from M.F. F.H. generated the Ad5ddTomato viral construct. M.G. provided critical reagents for the research. P.B. wrote the manuscript with contributions from M.F. and M.G. The research was overseen by M.F., M.G., and V.C. with funding acquisition by V.C. All authors discussed experimental results and contributed to the review and editing of the manuscript, providing approval of the final version of the manuscript.

## Declaration of interests

The authors declare no competing interests.

## Supplemental information

Document S1. Figure S1

Document S2. Article plus supplemental information
