# Supplementary material for: An automated and high-throughput approach for enhanced precision of adenoviral titering
Source: Mol Ther Methods Clin Dev. 2025 Jan 20;33(1):101410. doi: 10.1016/j.omtm.2025.101410 (PMC11834061; doi:10.1016/j.omtm.2025.101410)
Supplement: Document S1. Figure S1 [file mmc1.pdf]

OMTM, Volume *33*

Supplemental information

An automated and high-throughput approach for enhanced precision of adenoviral titering

Paolo Bottega, Manlio Fusciello, Firas Hamdan, Jacopo Chiaro, Salvatore Russo, Federica D'Alessio, Mikaela Grönholm, and Vincenzo Cerullo

Supplemental Material

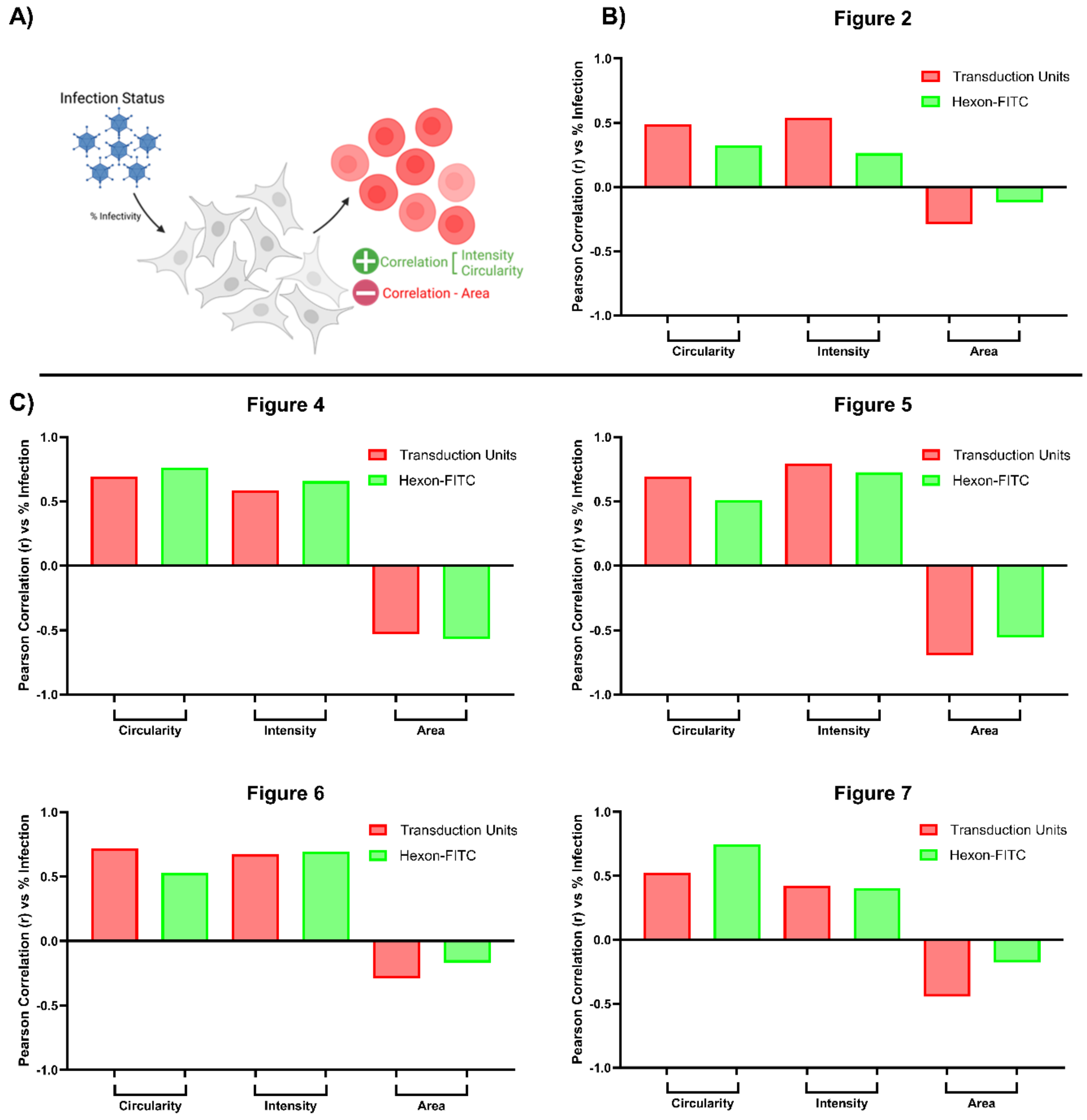

Figure S1: Pearson Correlations of Measurement Metrics. Statistical analysis of the correlation between the new protocol specific metrics (circularity, intensity, area) and the status of infection (% infectivity). A) Schematic representation of the correlations being highlighted throughout the figures. As the infection status increases, the fluorescent intensity and circularity of infected cells are observed to increase while the 2-dimensional area of the cells decrease. Image created via BioRender. B) Unranked waterfall plot of Pearson correlations

between infection status and the aforementioned metrics for Figures 2. C) Unranked waterfall plots of Pearson correlations between infection status and the new metrics for Figures 4 - 7 across varying time points.
